# Osteocalcin does not influence acute or chronic inflammation in human vascular cells

**DOI:** 10.1002/jcp.29231

**Published:** 2019-09-24

**Authors:** Sophie A. Millar, Ieva Zala, Susan I. Anderson, Saoirse E. O'Sullivan

**Affiliations:** ^1^ Division of Graduate Entry Medicine and Medical Sciences, School of Medicine, Royal Derby Hospital University of Nottingham Derby UK

**Keywords:** bone hormone, endothelial, inflammation, osteocalcin, smooth muscle

## Abstract

Some human observational studies have suggested an anti‐inflammatory role of osteocalcin (OCN). An inflammatory protocol using interferon‐γ and tumor necrosis factor‐α (10 ng/ml) was employed to examine the acute (24 hr) and chronic (144 hr) effects of uncarboxylated OCN (ucOCN) in commercial, primary, subcultured human aortic endothelial cells (HAEC), and human smooth muscle cells (HASMCs). The inflammatory protocol increased phosphorylation of intracellular signaling proteins (CREB, JNK, p38, ERK, AKT, STAT3, STAT5) and increased secretion of adhesion markers (vascular cell adhesion molecule‐1, intracellular adhesion molecule‐1, monocyte chemoattractant protein‐1) and proinflammatory cytokines (interleukin‐6 [IL‐6], IL‐8). After acute inflammation, there were no additive or reductive effects of ucOCN in either cell type. Following chronic inflammation, ucOCN did not affect cell responses, nor did it appear to have any pro‐ or anti‐inflammatory effects when administered acutely or chronically on its own in either cell type. Additionally, ucOCN did not affect lipopolysaccharide (LPS)‐induced acute inflammation in HAECs or HASMCs. The findings of this study do not support a causal role for OCN within the models of vascular inflammation chosen. Further confirmatory studies are warranted.

## Graphical abstract

The effects of uncarboxylated osteocalcin (OCN) was examined in acute and chronic models of inflammation in human aortic endothelial and smooth muscle cells. The addition of OCN alongside the inflammatory protocols did not affect inflammation as measured by secretory cytokines, adhesion molecules, and intracellular signaling.

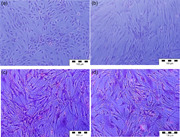

## 1 INTRODUCTION

Atherosclerosis is a progressive inflammatory disease, involving revolving phases of inflammation leading to macrophage infiltration and microcalcifications (Hutcheson & Aikawa, 2014; Ross, 1999). Inflammatory cytokines, chemoattractant proteins, and adhesion molecules are present throughout the atherosclerotic process, of which the endothelium and smooth muscle cell layers are both pivotal. The process of atherosclerotic calcification is also crucially triggered by inflammatory‐related pathways (Bessueille & Magne, 2015; Mazzini & Schulze, 2006). A paradox vascular‐bone axis exists as while bone formation decreases with age, biomineralization of the vasculature is increased, which is suggested to be due to a shared etiology of inflammation (Bessueille & Magne, 2015; Sage, Tintut, & Demer, 2010). The endocrine function of bone, particularly osteocalcin (OCN), is increasingly under investigation due to its link with whole body metabolism and far‐reaching extra‐skeletal effects (Lee et al., 2007; Oldknow, Macrae, & Farquharson, 2015; Oury et al., 2011; Oury et al., 2013).

OCN is produced predominantly by osteoblasts and is the most abundant, noncollagenous protein found in the mineralized matrix of bone (Hauschka, Lian, & Gallop, 1975). Posttranslational γ‐carboxylation of three glutamic acid residues within OCN results in carboxylated OCN (cOCN). However, OCN can be carboxylated to varying degrees, allowing for undercarboxylated forms to be present in the circulation (one or two carboxylated residues), and uncarboxylated OCN (ucOCN; no carboxylated residues). ucOCN has previously been regarded as the “active form,” and it has less affinity to bind to hydroxyapatite crystals in bone than cOCN.

Cross‐sectional observational studies in humans have demonstrated inverse associations between OCN and systemic inflammatory markers, such as C‐reactive protein (CRP) and interleukin‐6 (IL‐6; Bao et al., 2013; Chen et al., 2013; Liao et al., 2015; Lucey et al., 2013; Pittas, Harris, Eliades, Stark, & Dawson‐Hughes, 2009; Sarkar & Choudhury, 2013). A study including 108 newly‐diagnosed Type 2 diabetic patients reported that total serum OCN levels were inversely associated with IL‐6 and CRP (Sarkar & Choudhury, 2013), these results were also evidenced in an older healthy population (*n* = 380; Pittas et al., 2009). Two large studies in Chinese males (*n* = 2,043 and *n* = 1,768) with metabolic syndrome or normal metabolic state also found an inverse association between total serum OCN and CRP (Bao et al., 2013; Liao et al., 2015). In young obese and overweight women, as well as in postmenopausal women, these findings have also been replicated (Lucey et al., 2013; Chen et al., 2013). It is also known that subclinical inflammation is associated with altered bone metabolism, which further potentiates a role for OCN within the vasculature during inflammation (Ding, Parameswaran, Udayan, Burgess, & Jones, 2008; Kim, Kim, & Sohn, 2010; Koh et al., 2005).

In white‐adipose tissue from ucOCN‐treated obese mice, several inflammatory genes and transcription factors were downregulated including tumor necrosis factor (TNF); IL‐1β; IL‐6; chemokine (C‐C motif) ligand, Ccl2; caspase 1; and NLR family, pyrin domain containing 3, Nlrp3 (Guedes, Esteves, Morais, Zorn, & Furuya, 2017). Within the same study, ucOCN‐treated (20 ng/ml) mouse adipocytes similarly displayed a reduction in expression of inflammatory genes following stimulation with TNF‐α (Guedes et al., 2017).

OCN has previously been shown to increase nitric oxide production and prevent free fatty acid‐induced apoptosis in human aortic endothelial cells (HAECs); increase angiogenesis in a chick embryo in vivo model; increase proliferation in HAECs and HASMCs; and increase glucose metabolism and promote vascular calcification in OCN‐over expressing mouse chondrocytes and vascular smooth muscle cells (Cantatore, Crivellato, Nico, & Ribatti, 2005; Idelevich, Rais, & Monsonego‐Ornan, 2011; Jung et al., 2013; Millar, Anderson, & O'sullivan, 2019). Interestingly, Hill et al. (2014) demonstrated an anti‐inflammatory role of both cOCN and ucOCN (20 ng/ml) in isolated rat adipocytes by decreasing TNF‐α secretion, while cOCN also decreased IL‐6 secretion (Hill et al., 2014). Furthermore, in whole tissue extracts, IL‐10 (an anti‐inflammatory cytokine) was increased (Hill et al., 2014).

The potential role for OCN in the development of atherosclerosis has been recently extensively reviewed (Tacey et al., 2018). The question remains however whether OCN is a pathological bystander or in fact mediator. To our knowledge, no in vitro studies to date have investigated the inflammatory role of OCN in human vascular cells. The aim of the current experiments was to investigate the distinct role of ucOCN in HAECs and human aortic smooth muscle cells (HASMCs) after acute and chronic administration, with and without stimulated inflammation. We hypothesized that ucOCN may reduce proinflammatory markers when administered on its own and may reduce the stimulated inflammatory responses of HAECs and HASMCs.

## 2 MATERIALS AND METHODS

### 2.1 Osteocalcin

Human ucOCN (amino acids 1–49, [Glu17,21,24]) was purchased from AnaSpec Inc. CA (AS‐65307). The amino acid sequence of purchased OCN was as follows: Tyr‐Leu‐Tyr‐Gln‐Trp‐Leu‐Gly‐Ala‐Pro‐Val‐Pro‐Tyr‐Pro‐Asp‐Pro‐Leu‐Glu‐Pro‐Arg‐Arg‐Glu‐Val‐Cys‐Glu‐Leu‐Asn‐Pro‐Asp‐Cys‐Asp‐Glu‐Leu‐Ala‐Asp‐His‐Ile‐Gly‐Phe‐Gln‐Glu‐Ala‐Tyr‐Arg‐Arg‐Phe‐Tyr‐Gly‐Pro‐Val. The same batch of ucOCN has been previously shown to be biologically active in vascular cells in our previous work (Millar et al., 2019).

### 2.2 Cell culture

HAECs and HASMCs were purchased from PromoCell (UK) and maintained at 37°C in a humidified incubator supplemented with 5% CO_2._ Cells were cultured in endothelial cell growth media and smooth muscle cell growth media, respectively, containing 1% penicillin–streptomycin (Sigma‐Aldrich, UK) and supplemental mix (PromoCell, UK). In all experiments, cells were used between passages 3 and 5. After experimental treatments, cell media was collected and cells were washed once with phosphate‐buffered saline (PBS; pH 7.4; Gibco™). Radioimmunoprecipitation buffer (Sigma‐Aldrich) with protease and phosphatase inhibitors (A32959; Thermo Fisher Scientific) was added to lyse the cells and the plates were shaken at 4°C for an hour. The cells were then collected and centrifuged at 14,000*g* for 5 min at 4°C and cell supernatants were frozen at −80°C.

### 2.3 Acute and chronic inflammation

Acute inflammation was induced by treating cells with 10 ng/ml interferon‐γ (IFN‐γ) or vehicle (ethanol) for 8 hr, followed by addition of 10 ng/ml TNF‐α or vehicle (1% ethanol) for 16 hr, as described previously (Alhamoruni, Wright, Larvin, & O'sullivan, 2012). Cells were cotreated with or without ucOCN (10 ng/ml; Hannemann et al., 2013; Hu et al., 2013; Withold, Friedrich, & Degenhardt, 1997) for 24 hr in the acute inflammation protocol. The chronic inflammatory protocol included 8 hr of IFN‐γ (5 ng/ml) followed by addition of TNF‐α (5 ng/ml). After 48 hr, the media was replaced and treatment repeated up until 144 hr. Cells were treated with or without ucOCN (10 ng/mL). Media was collected at each time point and stored at −80°C until analyzed.

To test whether ucOCN could effect the production of endogenously induced inflammation, in a subset of experiments, lipopolysaccharide (LPS; 10 ng/ml) was added to HAECs and HASMCs for 24 hr with and without ucOCN (10 ng/ml). Media was collected after this time and stored at −80°C until analyzed.

### 2.4 Total protein content

A bicinchoninic acid protein assay was performed to quantify the total protein content in the cell lysates collected at the end of the experiments (Smith et al., 1985).

### 2.5 Cell signaling assay

The MILLIPLEX MAP 9‐plex Multi‐Pathway Magnetic Bead Signaling Kit (catalog no. 48‐680MAG; Merck Millipore) was performed per the manufacturers’ instructions to detect changes in phosphorylated ERK/MAP kinase 1/2 (Thr185/Tyr187), AKT (Ser473), STAT3 (Ser727), JNK (Thr183/Tyr185), p70s6 kinase (Thr412), NFkB (Ser536), STAT5A/B (Tyr694/699), CREB (Ser133), and p38 (Thr180/Tyr182) in cell lysates using the Luminex® xMAP® technology (48‐680MAG; Milliplex™; Merck Millipore).

### 2.6 Enzyme‐linked immunosorbent assays

Total OCN, endothelin pan specific, intracellular adhesion molecule‐1 (ICAM‐1)/CD54, vascular cell adhesion molecule‐1 (VCAM‐1)/CD106, total MMP‐3, CCL2/monocyte chemoattractant protein‐1 (MCP‐1), IL‐8/CXCL8, and IL‐17 DuoSet enzyme‐linked immunosorbent assays (ELISA) were performed on cell culture media as per the manufacturer's instructions (catalog no. DY1419; R&D systems, DY1160, DY720, DY809, DY513, DY279, DY208, and DY317). IL‐6 ELISA Ready‐SET‐Go! was performed on cell culture media as per the manufacturer's instructions (catalog no. 88‐7066‐22; Affymetri; eBioscience).

### 2.7 Lactate dehydrogenase activity assay

Lactate dehydrogenase activity (LDH) Colorimetric Assay kit (category no. ab102526; Abcam) was performed on cell media from the chronic inflammation experiment as per manufacturer's instructions.

### 2.8 Haematoxylin and eosin staining

After 48 hr of the chronic inflammation protocol, a representative selection of HAECs and HASMCs was washed with PBS and fixed with ice cold methanol/acetone (50:50) for 10 min at room temperature. Fixed cells were then washed with PBS and stained with 0.1% Mayer's haematoxylin and counterstained with 1% eosin Y solution to allow visualization of the nuclei and cytoplasm.

### 2.9 Statistical analysis

One‐way analysis of variances (ANOVAs) were performed to assess differences in protein secretions and protein phosphorylation after 24 hr. Data were mostly normally distributed and nonparametric *t* tests (Mann–Whitney) were performed in a few cases were the data were not normalized. Results were normalized to protein content. Two‐way ANOVAs were performed to detect differences in IL‐6 secretion and LDH activity using time and treatment as factors for the chronic inflammation experiment. Multiple comparisons were adjusted for by Dunnett's statistical hypothesis test. All statistical analyses were performed using Prism 7 for Windows (Version 7.01; GraphPad Software Inc.). *p* Values were considered significant at *p* < .05.

## 3 RESULTS

### 3.1 HAEC responses to acute OCN

**Figure 1:**
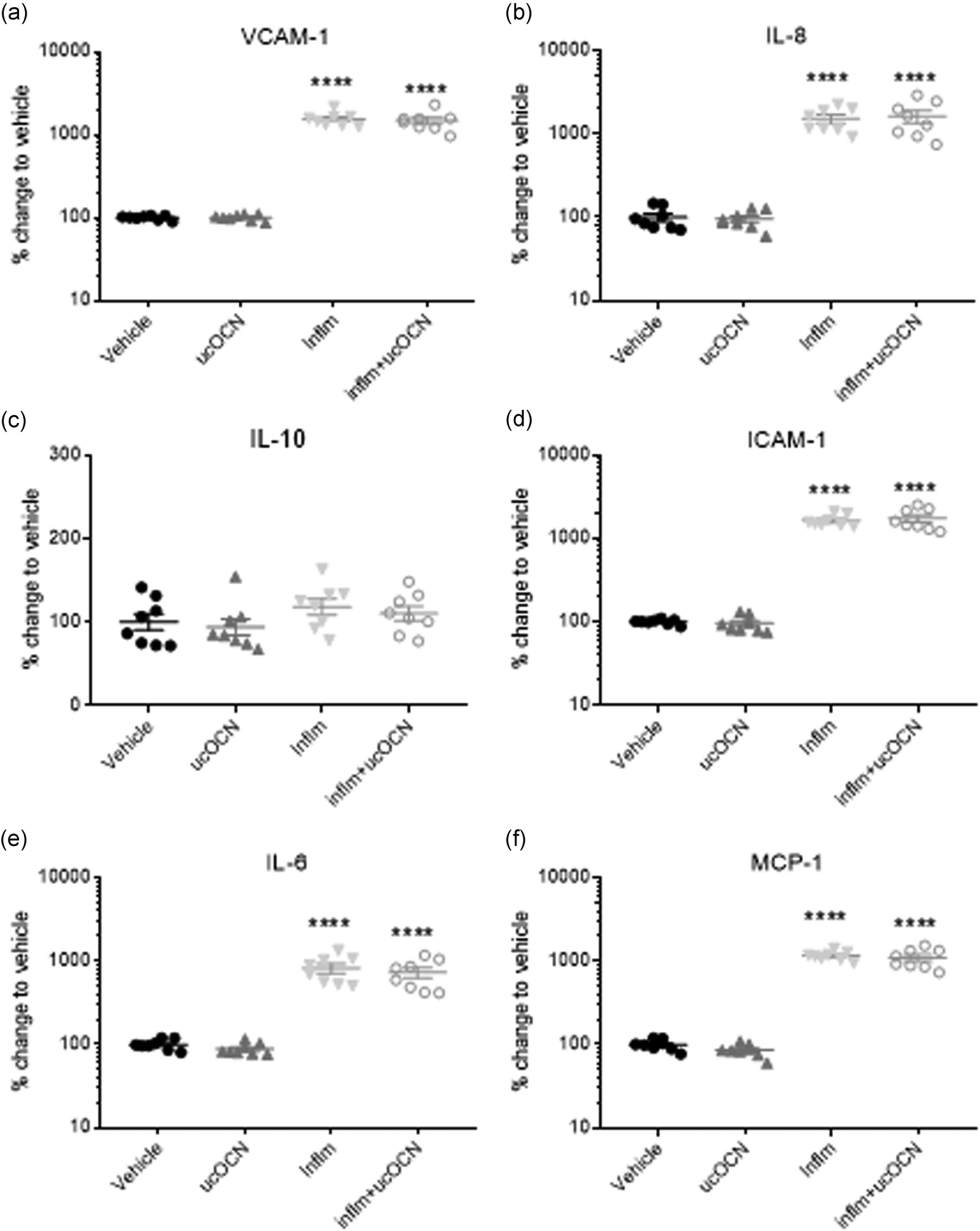
Protein secretion responses in HAECs after acute inflammation. Mean % change relative to vehicle and *SEM* of secreted cell proteins VCAM‐1, ICAM‐1, IL‐6, endothelin, IL‐10, IL‐8, and MCP‐1 when treated with vehicle, ucOCN (10 ng/mL), an inflammatory protocol (IFN‐γ and TNF‐α, both 10 ng/ml, 8 hr followed by 16 hr, respectively), or inflammatory protocol and ucOCN. Data were normalized to total protein content. Data were analyzed by one‐way ANOVA. **** indicates *p *< .001 compared with vehicle. HAEC, human aortic endothelial cell; ICAM‐1, intracellular adhesion molecule‐1; IL, interleukin; inflm, inflammatory protocol; MCP‐1, monocyte chemoattractant protein‐1; ucOCN, uncarboxylated osteocalcin; *SEM*, standard error of mean; VCAM‐1, vascular cell adhesion molecule‐1

**Figure 2:**
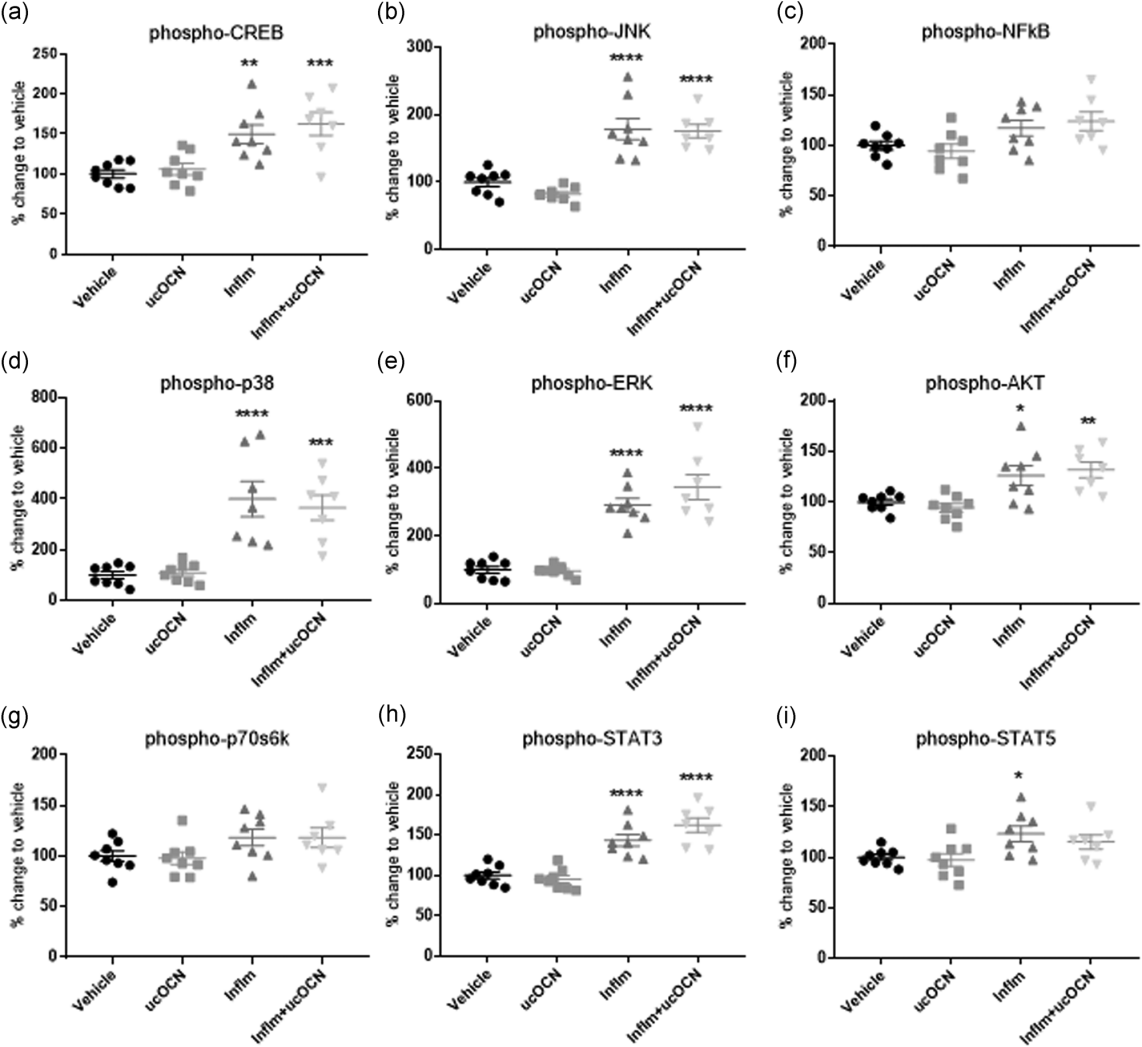
Intracellular signaling responses in HAECs. Luminex® xMAP® technology was used to detect changes in phosphorylated CREB (pS133), JNK (pT183/pY185), NFkB (pS536), p38 (pT180/pY182), ERK (pT185/pY187), Akt (pS473), p70 S6K (pT412), STAT3 (pS727), and STAT5A/B (pY694/699; 48‐680MAG; Milliplex™; Merck Millipore) in cell lysates when treated with vehicle or ucOCN (10 ng/ml) with and without inflammatory stimulus (IFN‐γ and TNF‐α) for 24 hr. Data were analyzed by one‐way ANOVA with multiple comparisons to vehicle corrected for by Dunnett's test. Data are given as means with error bars representing *SEM*. *denotes a significant difference compared with vehicle (*p < .05. **p < .01, ***P < .005, ****p < .001). HAEC, human aortic endothelial cell; IFN‐γ, interferon‐γ; inflm, inflammatory protocol (8 hr of IFN‐γ 10 ng/ml followed by addition of TNF‐α 10 ng/mL for 16 hr); *SEM*, standard error of mean; TNF‐α, tumor necrosis factor‐α; ucOCN, uncarboxylated osteocalcin

No changes in protein secretions (VCAM‐1, IL‐8, ICAM‐1, IL‐10, IL‐6, or MCP‐1) were observed in response to ucOCN in HAECs after 24 hr (Figure 1a–f). Furthermore, total protein content levels did not differ between ucOCN and vehicle (Figure S1a). No intracellular signaling protein phosphorylation (CREB, NFkB, p38, JNK, AKT, ERK, STAT3, STAT5, or p70s6k) was affected by ucOCN (Figure 2a–i).

### 3.2 HAEC responses to acute inflammation

Acute inflammation significantly increased the secretion of ICAM‐1, VCAM‐1, IL‐6, IL‐8, and MCP‐1 (*p* < .001; Figure 1a,b,d–f). IL‐10 secretion was not affected (Figure 1c). Phosphorylation of CREB, JNK, p38, ERK, AKT, STAT3, and STAT5 was increased with inflammation (Figure 2a,b,d–f,h,i). Phosphorylation of NFkB and p70s6k was not affected (Figure 2c,g). There were no significant differences in cell signaling (CREB, p38, JNK, ERK, AKT, STAT3, STAT5, NFkB, p70s6k), protein secretion, or total protein content between cells treated with or without ucOCN alongside inflammation after 24 hr.

### 3.3 Human aortic endothelial cell responses to chronic OCN

**Figure 3:**
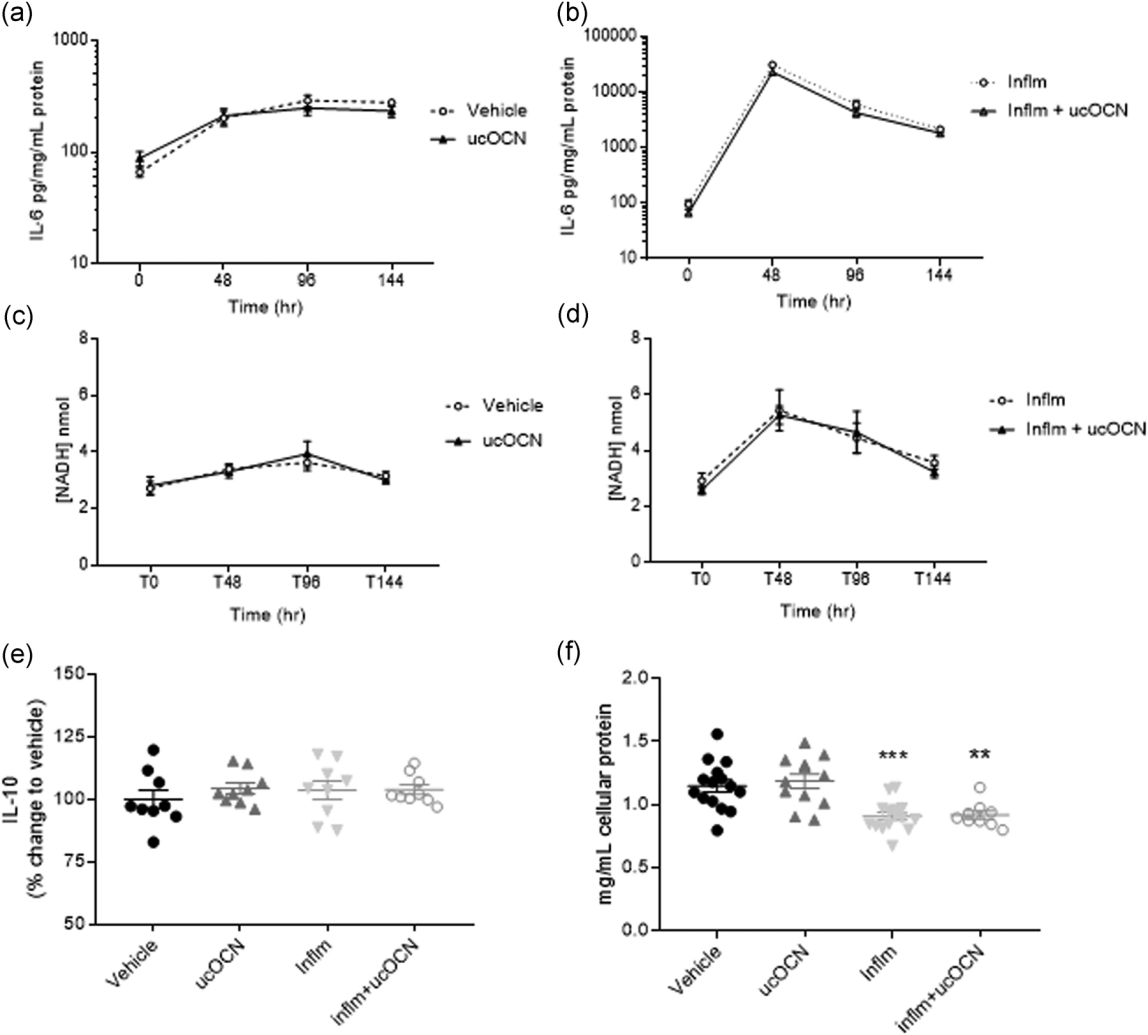
IL‐6 secretion, LDH activity, IL‐10 secretion, and total protein content in HAECs after chronic inflammation with or without OCN. The effects of ucOCN on secretion of IL‐6 without inflammation (a) and with inflammation (b) after 0, 48, 96, and 144 hr. The effect of ucOCN on LDH activity was measured by NADH concentration without inflammation (c) and with inflammation (d) after 0, 48, 96, and 144 hr. IL‐10 secretion was measured after 144 hr (e). Total protein content was measured by BCA assay at the end of the experiment. ucOCN (10 ng/ml) was added every 48 hr. Total *n* = 9 from three experimental repeats. Data are given as means with error bars representing *SEM*. *denotes a significant difference compared with vehicle, analyzed by one‐way ANOVA (**p < .01, ***p < .005). ANOVA, analysis of variance; HAEC, human aortic endothelial cell; IL, interleukin; IFN‐γ, interferon‐γ; inflm, inflammatory protocol (8 hr of IFN‐γ followed by addition of TNF‐α; both 5 ng/ml); LDH, lactate dehydrogenase; NADH, nicotinamide adenine dinucleotide H; *SEM*, standard error of mean; TNF‐α, tumor necrosis factor‐α; ucOCN, uncarboxylated osteocalcin

In the chronic experiments, IL‐6 secretion increased over time but was not affected by OCN (10 ng/ml) after 48, 96, or 144 hr incubation (Figure 3a). Similarly, no effects of ucOCN were observed on LDH activity, IL‐10 secretion, or total protein content (Figure 3c–f).

### 3.4 Human aortic endothelial cell responses to chronic inflammation

**Figure 4:**
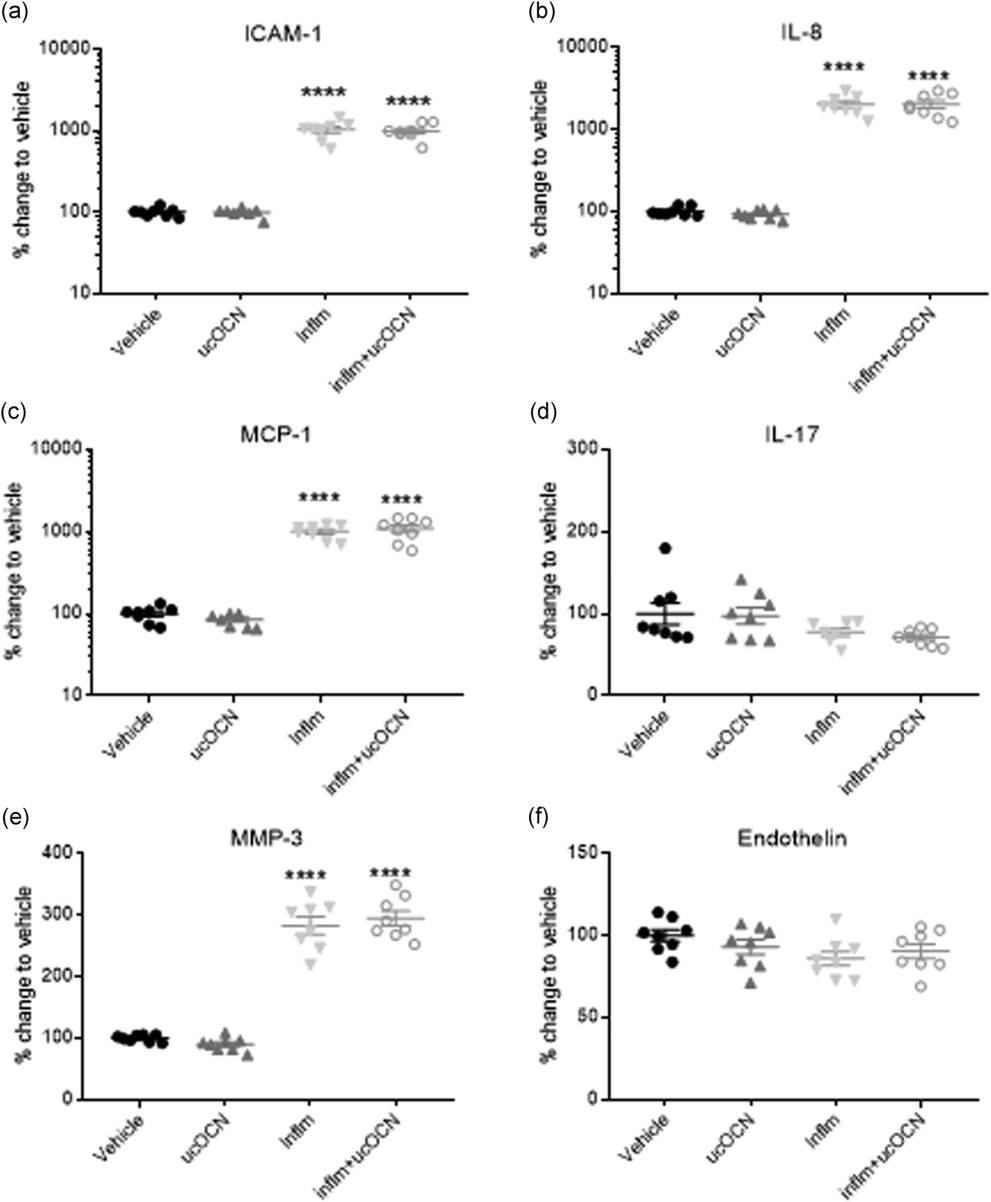
Protein secretions in HASMCs after acute inflammation. Mean % change relative to vehicle and *SEM* of secreted cell proteins ICAM‐1, MCP‐1, endothelin, MMP‐3, IL‐17, and IL‐8 when treated with vehicle, ucOCN (10 ng/ml), an inflammatory protocol (IFN‐γ and TNF‐α, both 10 ng/ml, 8 hr followed by 16 hr, respectively), or inflammatory protocol and ucOCN. Data were normalized to total protein content. Data were analyzed by one‐way ANOVA. ****denotes a significant difference compared with vehicle (*p* < .005). ANOVA, analysis of variance; HASMC, human aortic smooth muscle cell; ICAM‐1, intracellular adhesion molecule‐1; IL, interleukin; IFN‐γ, interferon‐γ; MCP‐1, monocyte chemoattractant protein‐1; MMP‐3, matrix metalloproteinase; *SEM*, standard error of mean; TNF‐α, tumor necrosis factor‐α; ucOCN, uncarboxylated osteocalcin; inflm, inflammatory protocol

Because we found no anti‐inflammatory effects of ucOCN in the acute inflammatory protocol, we next established whether any effects become apparent after prolonged inflammation and ucOCN treatment. IL‐6 secretion increased over time with the inflammatory protocol, with a peak observed at 48 hr, but ucOCN demonstrated no additional affects (Figure 3b). Nicotinamide adenine dinucleotide H concentration was measured as an indicator of LDH activity. LDH activity increased sharply following 48 hr of inflammation in HAECs, following a similar trajectory as with IL‐6 production (Figure 4d). There were no differences detected between cells treated with ucOCN alongside inflammation and those treated with inflammation alone (Figure 3d). IL‐10 secretion at 144 hr did not differ between treatment groups (Figure 3e). Total protein content (as an indicator of cell death) was decreased with the inflammatory protocol (*p* < .05) but no additional effect of ucOCN was observed (Figure 3f).

After 48 hr of inflammatory protocol, HAECs adopted an activated, spindle‐shaped morphology as opposed to the characteristic cobblestone appearance of noninflamed cells (Figure S2).

### 3.5 HASMC responses to acute OCN

**Figure 5:**
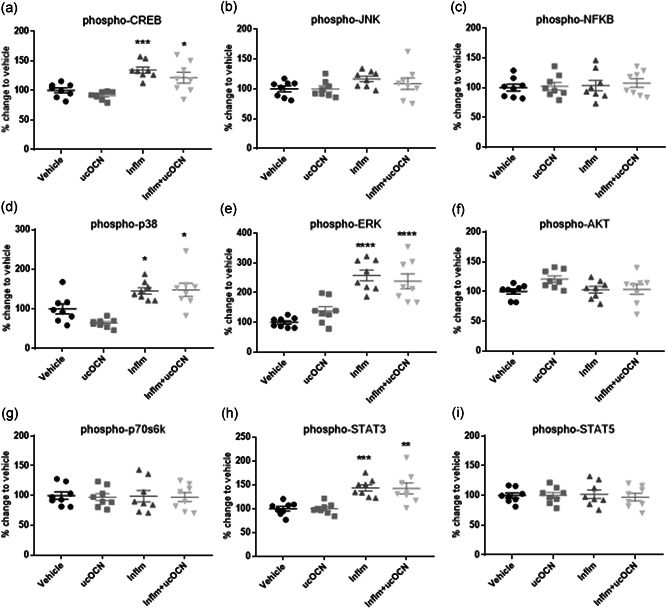
Intracellular signaling responses in HASMCs. Luminex® xMAP® technology was used to detect changes in phosphorylated CREB (pS133), JNK (pT183/pY185), NFkB (pS536), p38 (pT180/pY182), ERK (pT185/pY187), Akt (pS473), p70 S6K (pT412), STAT3 (pS727), and STAT5A/B (pY694/699; 48‐680MAG; Milliplex™; Merck Millipore) in cell lysates when treated with vehicle or ucOCN (10 ng/ml) with and without inflammatory stimulus (IFN‐γ and TNF‐α) for 24 hr. Data were analyzed by one‐way ANOVA with multiple comparison to vehicle corrected for by Dunnett's test. Data are given as means with error bars representing *SEM*. *denotes a significant difference compared with vehicle (*p < .05. **p < .01, ***p < .005, ****p < .001). ANOVA, analysis of variance; HASMC, human aortic smooth muscle cell; IFN‐γ, interferon‐γ; inflm, inflammatory protocol (8 hr of IFN‐γ 10 ng/ml followed by addition of TNF‐α 10 ng/ml for 16 hr); *SEM*, standard error of mean; TNF‐α, tumor necrosis factor‐α; ucOCN, uncarboxylated osteocalcin

No changes were observed in the secretion of ICAM‐1, IL‐8, MCP‐1, IL‐17, MMP‐3, or endothelin after 24 hr treatment with ucOCN compared with vehicle (Figure 4a–f). Phosphorylation of p38, ERK, AKT, CREB, JNK, NFkB, p70s6k, STAT3, and STAT5 was not affected by ucOCN exposure (Figure 5a–i). Total protein content did not differ with ucOCN treatment (Figure S1B).

### 3.6 HASMC responses to acute inflammation

ICAM‐1, MCP‐1, IL‐8, and MMP‐3 secretion was significantly increased by inflammation in HASMCs compared to vehicle (*p* < .001), however no effect of ucOCN was observed (Figure 4a–c,e). There was no significant effect of inflammation nor OCN on endothelin production or IL‐17 secretion (Figure 4d,f). Phosphorylation of CREB, p38, ERK, and STAT3 was increased with inflammation but no additional effect of ucOCN was observed (Figure 5a–i).

### 3.7 HASMC cell responses to chronic OCN

**Figure 6:**
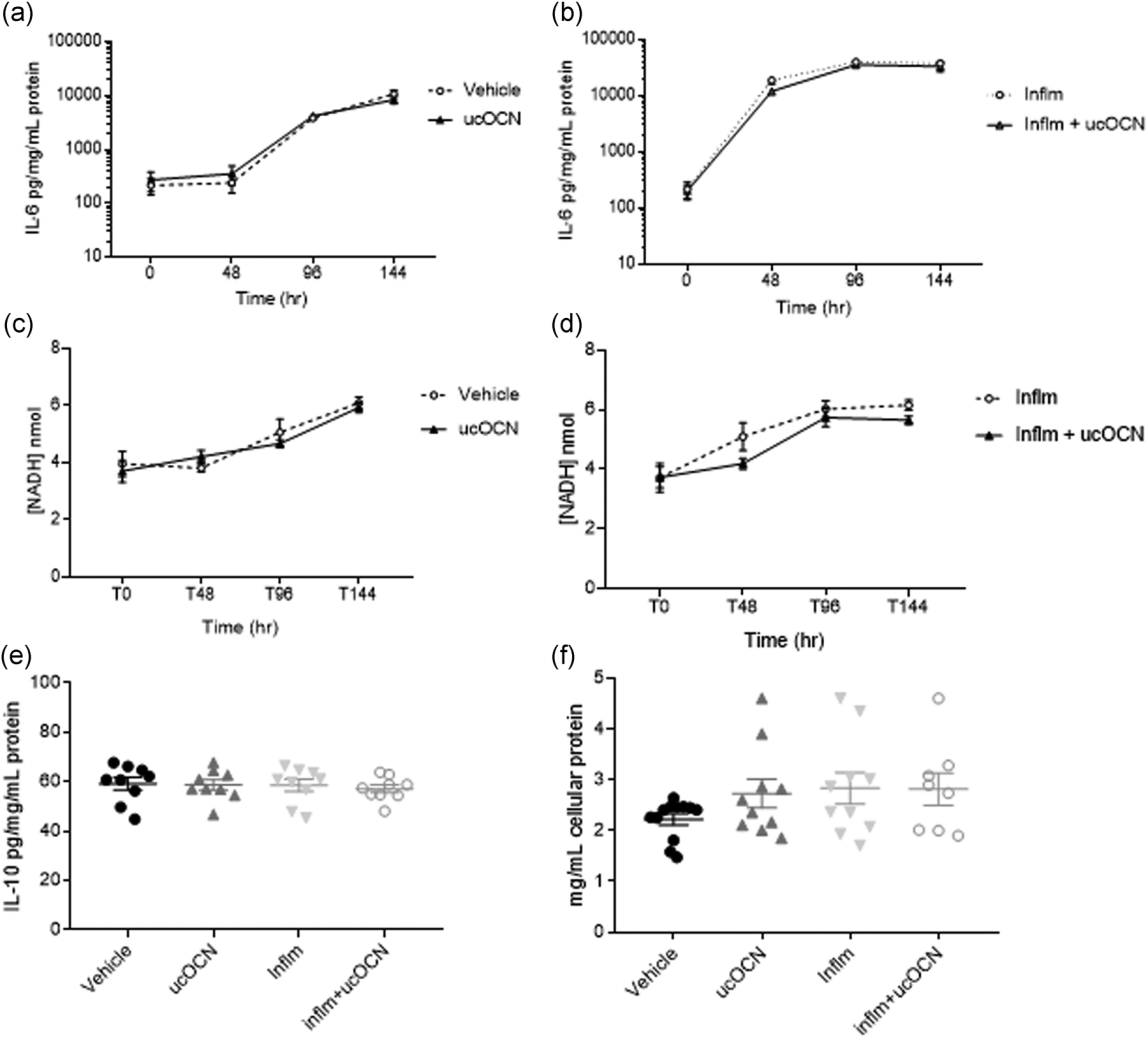
IL‐6 secretion, LDH activity, IL‐10 secretion, and total protein content in HASMCs after chronic inflammation with or without OCN. The effects of ucOCN on secretion of IL‐6 without inflammation (a) and with inflammation (b) after 0, 48, 96, and 144 hr. The effect of ucOCN on LDH activity was measured by NADH concentration without inflammation (c) and with inflammation (d) after 0, 48, 96, and 144 hr. IL‐10 secretion was measured after 144 hr (e). Total protein content was measured by BCA assay at the end of the experiment. ucOCN (10 ng/ml) was added every 48 hr. Total *n* = 9 from three experimental repeats. BCA, bicinchoninic acid; HASMC, human aortic smooth muscle cell; LDH, lactate dehydrogenase; IFN‐γ, interferon‐γ; IL, interleukin; inflm, inflammatory protocol (8 hr of IFN‐γ followed by addition of TNF‐α; both 5 ng/ml); NADH, nicotinamide adenine dinucleotide H; TNF‐α, tumor necrosis factor‐α; ucOCN, uncarboxylated osteocalcin

In the chronic experiments, IL‐6 secretion increased over time but was not affected by OCN after 48, 96, or 144 hr incubation (Figure 6a). LDH activity increased over time, with no additional effect of ucOCN observed (Figure 6c). Similarly, IL‐10 secretion and total protein content was not affected by OCN after 144 hr (Figure 6e,f).

### 3.8 HASMC responses to chronic inflammation

Following chronic exposure to inflammation, IL‐6 secretion increased in HASMCs until 96 hr and remained elevated (Figure 6b). Addition of ucOCN did not affect IL‐6 levels. Similarly, LDH activity was increased with inflammation but was not affected by the coadministration of ucOCN (Figure 6d). IL‐10 and total protein content were not altered by the inflammation protocol nor ucOCN (Figure 6e,f).

### 3.9 OCN secretion is not detected following chronic inflammation

Finally, total OCN was measured in cell media after chronic inflammation (144 hr) to test whether inflammation induced OCN secretion. OCN was not detectable in media from HAECs nor HASMCs at the end of the experiments (data not shown).

### 3.10 OCN does not prevent LPS‐induced inflammation

As ucOCN did not affect inflammation induced by exogenously added cytokines, we then tested whether LPS‐induced inflammation elicited a different response. Acute LPS treatment (24 hr) significantly increased secretion of VCAM‐1 and ICAM‐1 in HAECs and HASMCs, respectively, compared with control (Figure 1c,d). In both HAECs and HASMCs, there were no differences in VCAM‐1 or ICAM‐1 secretion respectively between LPS treated and LPS + ucOCN‐treated cells (Figure 1c,d).

## 4 DISCUSSION

OCN is synthesized predominantly by osteoblast cells, which acts as an extra‐skeletal hormone known to effect insulin sensitivity, insulin secretion, energy metabolism, cognition, and fertility (Lee et al., 2007; Oury et al., 2011; Oury et al., 2013). Population‐based cross‐sectional studies have reported a negative or inverse association between OCN and markers of systemic inflammation, such as IL‐6 and CRP, suggesting a potential anti‐inflammatory role (Chen et al., 2013; Kim et al., 2010; Liao et al., 2015; Pittas et al., 2009; Sarkar & Choudhury, 2013; Schett et al., 2006). However, the in vitro effects of OCN on inflammation within the vasculature have not yet been described. Therefore, we decided to try and reveal mechanisms of action of ucOCN during inflammation, as well as investigate the effects of acute and chronic administration of ucOCN on its own.

Here, we demonstrate for the first time using two human vascular cell types that ucOCN does not regulate or alter the inflammatory responses of HAECs or HASMCs during acute inflammation. Our observational results presented here do not reflect an anti‐inflammatory role of OCN suggested by some cross‐sectional, human studies, at least within the experimental models used. ucOCN did not affect inflammatory cytokine production nor inflammatory signaling pathways in HASMCs and HAECs. This may be representative of the nonbinary nature of OCN which cannot be limited to having a positive or negative influence overall in vascular pathology and physiology in humans. It may transpire that OCN plays a protective role in the later stages of atherosclerosis and have more of an impact on the process of calcification.

As no overall anti‐inflammatory effects of OCN were reported in the acute inflammatory protocol, we next established whether any effects of OCN become apparent after prolonged inflammation and OCN treatment. Pro‐ and anti‐inflammatory cytokine production as well as LDH activity were not affected by chronic ucOCN treatment, alone or in combination with the inflammatory protocol. Lastly, we further showed that in another model of inflammation using LPS, ucOCN did not affect VCAM‐1 or ICAM‐1 secretion in HAECs and HASMCs respectively. An interesting observation from this study was the transition of HAECs after 48 hr of chronic inflammation to an activated morphology that is characteristic of endothelial cells under sheer stress and inflammation, representative of the early stages of atherosclerosis (Hunt & Jurd, 1998). This corresponds to the upregulation of activated endothelium markers VCAM‐1 and ICAM‐1 reported at 24 hr. Additionally we demonstrated that inflammation does not induce secretion of OCN itself.

The role of OCN in cardiovascular disease has had conflicting results in humans. Some longitudinal studies have reported U‐shaped associations between OCN and cardiovascular‐related or all‐cause mortality (Kanazawa, Yamaguchi, & Sugimoto, 2011; Yang et al., 2017; Yeap et al., 2012). However, other studies have found no association between OCN and cardiovascular disease risk (Holvik et al., 2014; Hwang et al., 2015). A systematic review and meta‐analysis on the association between OCN and markers of atherosclerosis or calcification in humans found no conclusive relationship (Millar, Patel, Anderson, England, & O'sullivan, 2017). Although a causal role of ucOCN in vascular inflammation was not identified in this study, further work should explore whether OCN may influence systemic inflammation via directly acting on immune cells. Additionally, the exploration of the possible biological activity of other forms of circulating OCN should not be disregarded. We recognize that phenotypic drift occurs with the isolation and subculturing of cells and we acknowledge this as a limitation of the current study. Further work should explore both the effect of OCN on intact vessels and on cells from different vascular beds including resistance and conduit vessels.

In conclusion, in our models of vascular inflammation, OCN did not display a role in the direct inflammatory responses of primary subcultured HAECs or smooth muscle cells in either an acute or chronic setting.

## FUNDING INFORMATION

This work was supported by the Biotechnology and Biological Sciences Research Council (grant number: BB/M008770/1).

## CONFLICT OF INTERESTS

The authors declare that there are no conflict of interests.

## AUTHOR CONTRIBUTIONS

S. O. S. and S. M. designed the experiments which were carried out by S. M. and I. Z. (I. Z. carried out the IL‐6 and L. D. H. measurements for the chronic experiments). S. A. and S. O. S supervised the project. S. M. wrote the manuscript with input from S. O. S. and S. A.

## Supporting information

Supplementary Figure 1. Total protein content after 24 hr was measured by a BCA assay in (a) human aortic endothelial cells (HAECs) and (b) smooth muscle cells (HASMCs). (c) VCAM‐1 secretion following 24 hr inflammation stimulated by LPS (10 ng/ml) with and without ucOCN (10 ng/ml) in HAECs. (D) ICAM‐1 secretion following 24 hr inflammation stimulated by LPS (10 ng/ml) with and without ucOCN (10 ng/ml) in HASMCs. Data were analysed by one‐way ANOVA. Data are given as means with error bars representing *SEM*. *denotes a significant difference compared to vehicle (*p<.05, **p<.01, ****p<.001). ucOCN, uncarboxylated osteocalcin; inflm, inflammatory protocol (8 hr of IFN‐γ 10 ng/ml followed by addition of TNF‐α 10 ng/ml for 16 hr); LPS, lipopolysaccharide; *SEM*, standard error of mean

Supplementary Figure 2. Representative images of hematoxylin and eosin (H&E) staining of human aortic endothelial cells (HAECs) in vitro after 48 hr (a) and human aortic smooth muscle cells (HASMCs) in vitro after 48 hr (c). Activated HAECs with altered morphology (b) and HASMCS with no morphological changes (d) after 48 hr of induced inflammation by addition of IFN‐γ and TNF‐α (both 5 ng/ml; 8 hr of IFN‐γ followed by addition of TNF‐α)

## Data Availability

The data that support the findings of this study are available from the corresponding author upon reasonable request.
