# Supplementary figures and images for: Osteocalcin does not influence acute or chronic inflammation in human vascular cells
Source: J Cell Physiol. 2019 Sep 24;235(4):3414–24. doi: 10.1002/jcp.29231 (PMC6972510; doi:10.1002/jcp.29231)

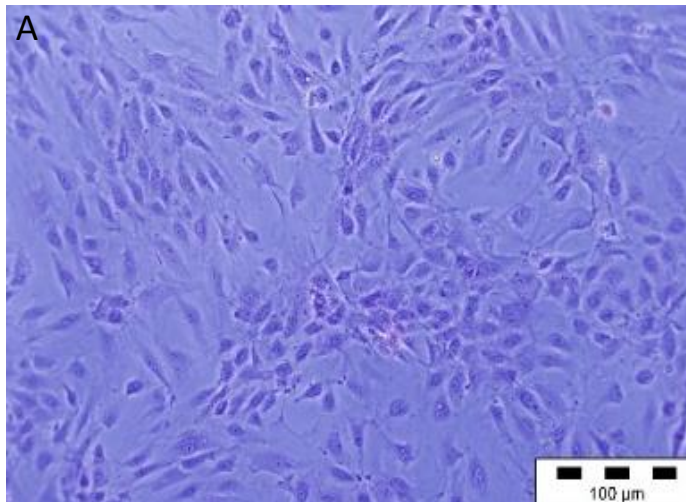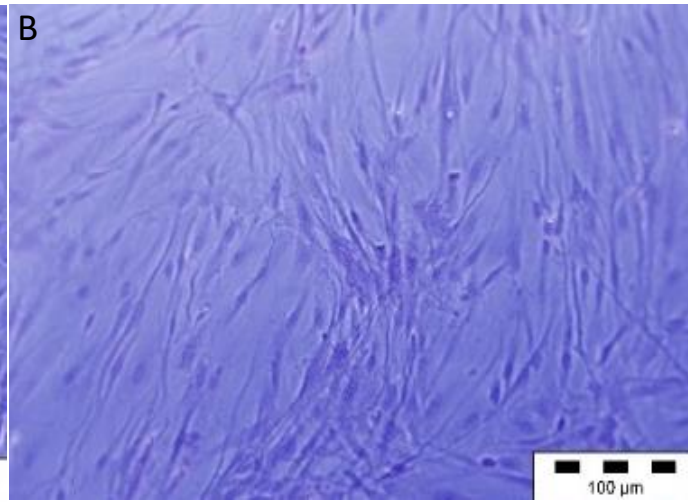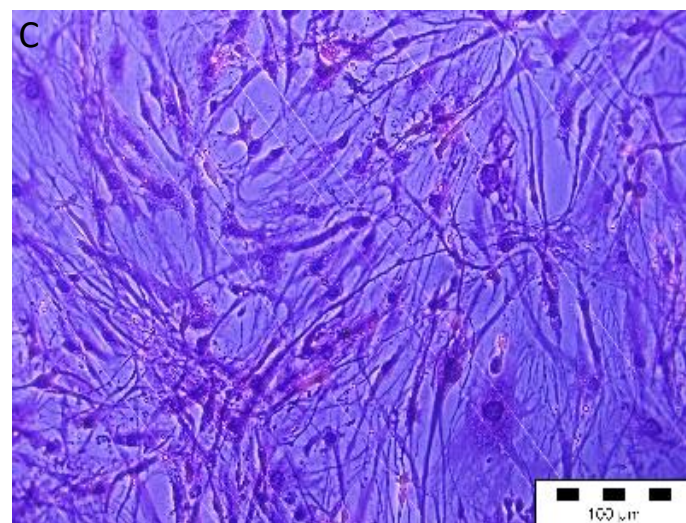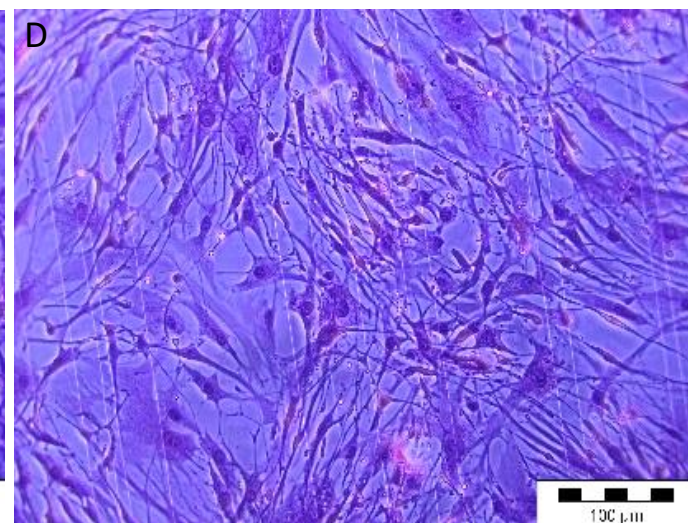

Supplement: Supplementary file 1 — Supplementary Figure 1. Total protein content after 24 hr was measured by a BCA assay in (a) human aortic endothelial cells (HAECs) and (b) smooth muscle cells (HASMCs). (c) VCAM‐1 secretion following 24 hr inflammation stimulated by LPS (10 ng/ml) with and without ucOCN (10 ng/ml) in HAECs. (D) ICAM‐1 secretion following 24 hr inflammation stimulated by LPS (10 ng/ml) with and without ucOCN (10 ng/ml) in HASMCs. Data were analysed by one‐way ANOVA. Data are given as means with error bars representing SEM. *denotes a significant difference compared to vehicle (*p<.05, **p<.01, ****p<.001). ucOCN, uncarboxylated osteocalcin; inflm, inflammatory protocol (8 hr of IFN‐γ 10 ng/ml followed by addition of TNF‐α 10 ng/ml for 16 hr); LPS, lipopolysaccharide; SEM, standard error of mean [file JCP-235-3414-s001.pdf]

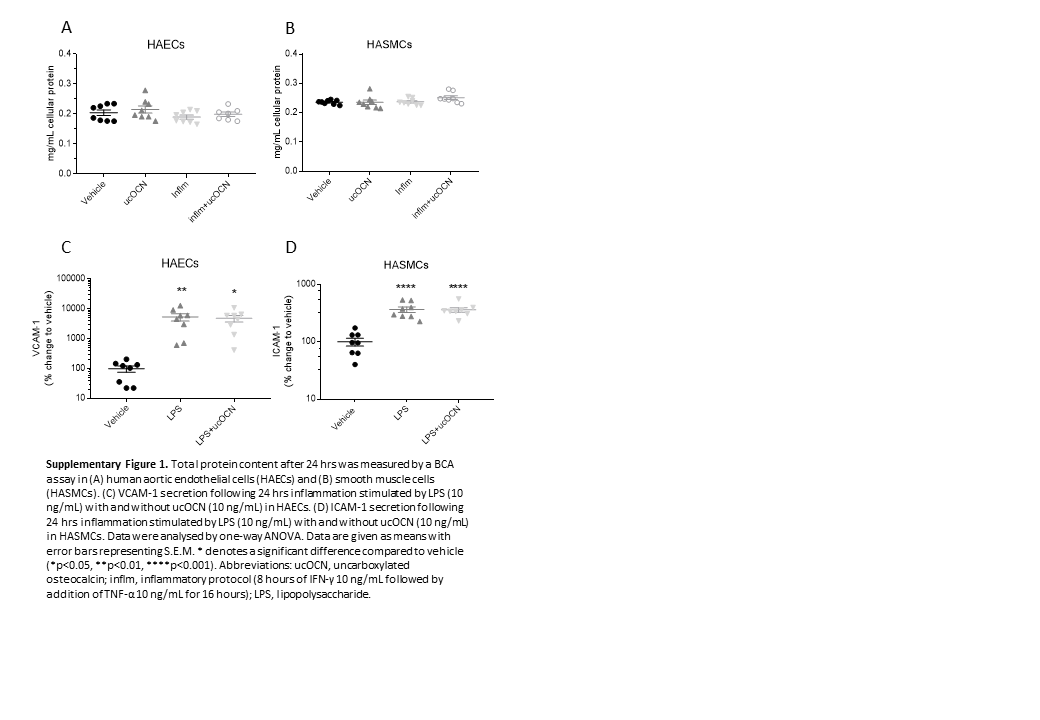

Supplement: Supplementary file 2 — Supplementary Figure 2. Representative images of hematoxylin and eosin (H&E) staining of human aortic endothelial cells (HAECs) in vitro after 48 hr (a) and human aortic smooth muscle cells (HASMCs) in vitro after 48 hr (c). Activated HAECs with altered morphology (b) and HASMCS with no morphological changes (d) after 48 hr of induced inflammation by addition of IFN‐γ and TNF‐α (both 5 ng/ml; 8 hr of IFN‐γ followed by addition of TNF‐α) [file JCP-235-3414-s002.TIF]
